# Efficacy of a school-based education intervention on the consumption of fruits, vegetables and carbonated soft drinks among adolescents

**DOI:** 10.1017/S1368980023002094

**Published:** 2023-12

**Authors:** Kazi R Ahmed, Tracy Kolbe-Alexander, Asaduzzaman Khan

**Affiliations:** 1 Department of Health Promotion and Health Education, Bangladesh University of Health Sciences, Darus Salam, Mirpur, Dhaka 1216, Bangladesh; 2 School of Health & Rehabilitation Sciences, The University of Queensland, Brisbane, Australia; 3 School of Health and Wellbeing, University of Southern Queensland, Toowoomba, Australia; 4 School of Human Movement and Nutrition Sciences, The University of Queensland, Saint Lucia, Australia; 5 Division of Exercise Science and Sports Medicine, Department of Human Biology, Faculty of Health Sciences, University of Cape Town, Rondebosch, South Africa

**Keywords:** Intervention, Adolescent, Nutrition education, Fruit, Vegetable, Carbonated soft drinks

## Abstract

**Objectives:**

To evaluate the efficacy of a school-based education intervention on the consumption of fruit, vegetables and carbonated soft drinks among adolescents.

**Design:**

Cluster-randomised controlled trial.

**Setting:**

Eight secondary schools from Dhaka, Bangladesh, participated in this trial and were randomly allocated to intervention (*n* 160) and control groups (*n* 160).

**Participants:**

A total of 320 students from 8th to 9th grades participated and completed the self-reported questionnaires at baseline, and at 8 and 12 weeks. The intervention included weekly classroom-based nutrition education sessions for students and healthy eating materials for students and parents. Repeated measures ANCOVA was used to assess the effects of the intervention.

**Results:**

Daily fresh fruit intake was more frequent in the intervention (26 %) compared to the control group (3 %) at 12 weeks (*p* = 0·006). Participants from the intervention group also reported a significantly (*P* < 0·001) higher (49 %) proportion of fresh vegetable intake compared to the control group (2 %) at 12 weeks. Frequency of daily carbonated soft drinks intake decreased (25 %) in the intervention group at 12 weeks compared to baseline, while it remained unchanged in the control group; the interaction effect was observed significant (*P* = 0·002).

**Conclusion:**

Our school-based education intervention increased the daily frequency of fresh vegetables and fruit intake and decreased carbonated soft drink consumption among adolescents in the intervention group. There is a need for scaling up the intervention to engage students and empower them to develop healthy dietary habits.

Healthy dietary behaviours can improve adolescents’ mental health and enhance cognitive skills (e.g. concentration and memory) and academic performance^(1)^. A healthy diet during adolescence reduces the risk of nutrition-related health problems, such as obesity, diabetes, Cardiovascular Disease (CVD), poor sleep patterns, depression and anxiety^(2,3)^. The transition from childhood into adolescence is often associated with unhealthy dietary behaviours^(4)^. Less than 30 % of adolescents in low-middle-income countries meet the WHO guidelines for fruit and vegetable intake^(5)^. Furthermore, many of low-middle-income countries have a double burden of malnutrition with a high prevalence of underweight and overweight which are resulted from nutritional deficiencies^(6,7)^. Dietary behaviours in low-middle-income countries are also rapidly changing as energy-dense, nutrient-poor foods become both available and affordable^(8)^.

Many low-middle-income countries, including Bangladesh, experienced a nutrition transition on rapid changes in diet consumption^(9)^. The dietary pattern of Bangladeshi adolescents is changing fast due to rapid urbanisation, economic growth and nutrition transition, particularly in the urban area^(10)^. Unhealthy dietary habits such as less intake of fruit and vegetable and a high consumption of carbonated soft drinks are prevalent among Bangladeshi adolescents^(11,12)^. The WHO recommends that adolescents consume at least five servings of fruit and vegetables a day^(13)^, which is also followed in Bangladesh. However, findings from the Global School-based Student Health Survey (GSHS) indicate that the majority of adolescents worldwide consume less than the recommended amount of fruit and vegetables, while their consumption of carbonated soft drinks tends to be higher^(14)^. In Bangladesh, only one-fifth of adolescents (21 %) reported eating five servings of fruit and vegetables a day^(15)^, while 47 % drank carbonated soft drinks one or more times per d^(16)^.

Schools are promising places in promoting intervention for healthy eating behaviours^(17)^. Systematic reviews indicated that school-based intervention is likely to be effective in increasing fruit and vegetable intake and decreasing carbonated soft drink consumption among adolescents^(18,19)^. Implementation of a Health Promoting Schools framework was able to encourage adolescents to adopt healthy eating behaviours and healthy food choices^(20)^. School-based interventions guided by the Health Promoting Schools framework, involving parents, teachers, interactive school activities and school environments, were likely to be more effective in enhancing healthy eating behaviours^(21)^. Moreover, multi-component school-based interventions through interactive classroom activities resulted in a significant improvement in eating more fruit and replaced high-calorie beverages with healthier alternatives among adolescents^(22,23)^. Furthermore, multi-component school-based educational interventions involving teachers and parents are likely to be more effective in increasing adolescents’ fruit and vegetable intake^(24)^. The purpose of this multi-component school-based intervention was to evaluate the efficacy of a 12-week education intervention on the consumption of fruit, vegetables and carbonated soft drinks among adolescents in Dhaka, Bangladesh.

## Methods

### Recruitment and participants

This study was conducted from March to June 2019 (prior to the COVID-19 outbreak) and used dietary outcomes of a cluster-randomised controlled trial, which was designed to promote physical activity and dietary behaviours and reduce sedentary behaviours. The trial was registered with the Australian and New Zealand Clinical Trials Registry (ACTRN12619000091101)^(25)^. A number of schools from different socio-economic levels were approached based on personal relationship with the research team, location and/or accessibility where the number of students in grades 8–9 at each school varied from 150 to 200.

The researcher (KRA) purposively selected and invited thirteen secondary schools in Dhaka, the capital city of Bangladesh, with schools being considered as clusters. Eleven schools expressed their willingness to participate, and eight schools were randomly allocated to represent the target population of this study. All randomly selected schools were then randomised into the intervention (*n* 4) and control schools (*n* 4) using computer-generated random numbers. The distance between schools (clusters) ranged from 2 to 7 kilometres, and hence, the chance of contamination was likely to be remote.

The sample size for this study was based on an assumption that the intervention will result in an increase of 10 min/d of moderate physical activity in the intervention group compared to the control group, as found in an earlier school-based intervention study^(26)^. A minimum sample of thirty-six students per cluster (i.e. school) was required to achieve 80 % power at a 5 % level of significance (one-tailed) for a standard deviation of 31·98 min and an assumed intra-cluster correlation coefficient of 0·03^(27)^. Considering attrition of 10 %, the required sample size was a total of forty students from each participating school (i.e. cluster). In the event of more than forty students in a selected school, a sample of forty students was randomly selected from each school, as per the inclusion criteria: (i) public and/or private high schools; (ii) located in the Dhaka city; and (iii) students from grade 8 and 9 (aged 13–17 years). Thus, a total of 320 students (160 per group; 4 per school/cluster) were included in the trial. There was no loss to follow-up in the trial.

### Intervention

The intervention was offered to all interested 8–9 grade students at the intervention schools. Students in the intervention schools received the programme components; students in the control schools received their usual curriculum during the intervention period. A 12-week multi-component intervention was developed by the research team based on the WHO’s Health Promoting Schools framework^(28)^ targeting the classroom component, school environment and parental involvement. A brief overview of these components is given as follows.

#### Classroom component

The present study was embedded in the school curriculum that offered the education session on physical activity, sedentary and healthy eating behaviours once a week during the physical education class by the researcher. Twelve education lessons including discussions and question-answer sessions were delivered in each intervention school. For example, students were asked to prepare classroom activities like ‘MyPyramid for Healthy Eating’ (see online Supplemental Fig. 1). The content of the intervention included healthy eating options (e.g. increasing intake of fruits and vegetables, fibre, and water and reducing consumption of snacks, fast foods, and avoiding sugary drinks), classification of food into the various food groups (e.g. carbohydrates, proteins, fats, vitamins, minerals, fibre and water), causes of overweight and obesity, health risks of overweight and obesity, and benefits of physical activity.

#### Schools’ environment

Teachers in the intervention school were requested to ask vendors around the school to stop selling unhealthy food. Schools and students were also given educational materials (such as infographics) with pictorial messages to encourage, enforce and stimulate healthy choices in the school public.

#### Parental involvement

The goal of the parent’s involvement was to create a supportive environment for healthy behaviours away from school and aware of their role in influencing their children’s dietary behaviours as well as to support their child in making healthy changes.

**Fig. 1:**
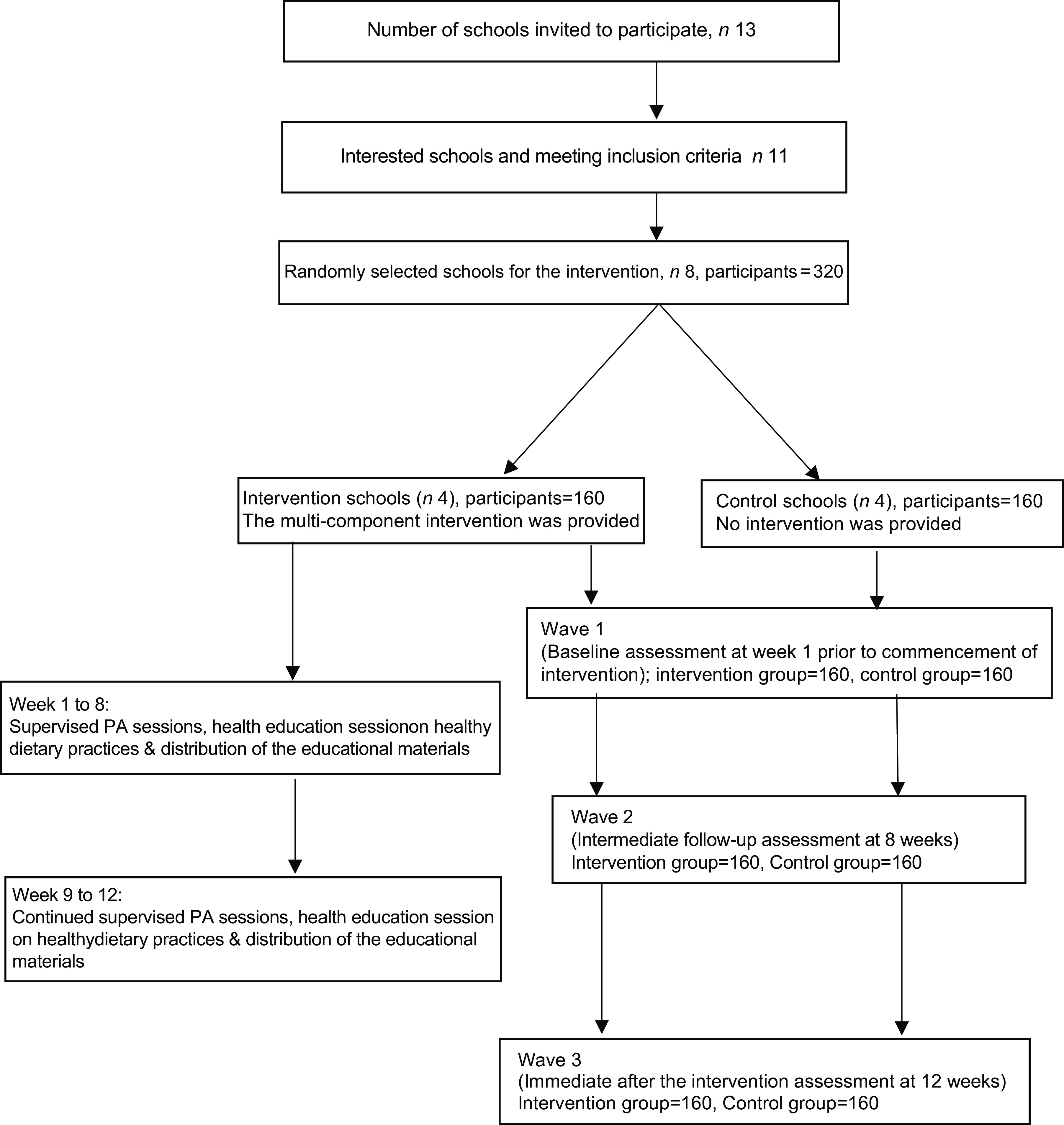
Flow diagram of the intervention illustrates an overview of the key components and order of activities involved in the intervention

Parents received eight infographics (e.g. see online Supplemental Fig. 2(a) and (b) for examples) with messages related to healthy foods and active lifestyles. Educational materials were designed as take-home messages to enhance students’ understanding and memory after the education session and build a link with their families. A flow diagram of the intervention study is presented in Fig. 1.

### Data collection procedure

Baseline (Wave 1) data, including anthropometric measurements, were collected before the intervention. The lead researcher (KRA) measured students’ height and weight. Height and weight were measured at baseline, 8 weeks (Wave 2) and 12 weeks (Wave 3). The weight and height were measured to the nearest 0·1 kg and 0·1 cm on a portable digital scale and a portable stadiometer, respectively^(29)^. We calculated BMI using Centers for Disease Control and Prevention (CDC) growth charts to group adolescents as underweight (BMI < 5th percentile), normal weight (BMI ≥ 5th to <85th percentile), overweight (BMI ≥ 85th to < 95th percentile) or obese (BMI ≥ 95th percentile)^(30)^. BMI percentiles were computed as a function of age (BMI-for-age) and transformed to a standard normal distribution (BMI-for-age *Z*-score) for using in modelling.

The students completed a self-administered questionnaire. Students were asked about their age, sex, grade, importance of physical activity, physical education classes and family-level data (e.g. parental education and family income data provided by parents), which were collected at baseline. Self-reported health (e.g. sleep disturbance and duration, physical and psychosocial health), dietary habits-related questions that included the daily frequency of fresh fruit, vegetables, and carbonated soft drink intakes, screen time and physical activity behaviours-related data were measured at baseline, 8 weeks and 12 weeks.

### Outcomes measures

Daily fruit and vegetable intake was measured separately using the relevant questions adapted from the GSHS^(31)^. Daily frequency of fresh fruit intake was assessed with a single question ‘During the past 30 d, how many times per day did you usually eat fruit, such as such as bananas, guava, mango, pineapple, apples, oranges, jackfruit, boroi, or amra?’ Prior to the study, the questionnaire was translated into the native language of the study participants. The response options for this question were ‘I did not eat fruit during the past 30 d’, ‘less than 1 time/d’, ‘1 time/d’, ‘2 times/d’, ‘3 times/d’, ‘4 times/d’ and ‘5 or more times/d’.

Daily frequency of fresh vegetable intake was assessed with a single question ‘During the past 30 d, how many times per day did you usually eat vegetables, e.g. potatoes, potol, cauliflower, cabbage, beans, brinjal, ladies’ finger, chichinga?’ ‘I did not eat vegetables during the past 30 d’, ‘less than 1 time/d’, ‘1 time/d’, ‘2 times/d’, ‘3 times/d’, ‘4 times/d’ and ‘5 or more times/d’^(31)^.

In the analysis, daily frequency of fresh fruit and vegetable intake was recoded to a continuous variable and categorised as 0 = did not eat, 0·5 =< 1 time/d, 1 = 1 time/d, 2 = 2 times/d, 3 = 3 times/d, 4 = 4 times/d and 5·5 = 5 or more times/d, respectively, as used elsewhere^(32)^. Daily frequency of carbonated soft drink intake was assessed with a single question ‘During the past 30 d, how many times per day did you usually drink carbonated soft drinks?’ ‘less than 1 time/d’, ‘1–2 times/d’, ‘3–4 times/d’, ‘5–6 times/d’, ’7–9 times/d’ and ‘10 or more times/d’^(31)^. Country-specific examples of carbonated soft drinks were provided (e.g. Coke, Fanta and 7Up), and the students were instructed not to include diet soft drinks. In the analysis, soft drink consumption was recoded to a continuous variable and categorised as 0 = none/d, 1·5 = 1–2 times/d, 3·5 = 3–4 times/d, 5·5 = 5–6 times/d, 8 = 7–9 times/d, and 11 = 10 or more times/d, respectively^(32)^.

### Explanatory variables

The explanatory variables included sex, age, BMI-based weight status, family-level data such as parental education and family income data provided by parents. A total recreational screen-time measure was derived by taking the weighted average (i.e. (school-day screen time × 5) + (weekend-day screen time × 2)/7)^(33)^. Physical activity levels were measured using the modified version of the International Physical Activity Questionnaire for Adolescents (IPAQ-A), which has been validated in European adolescents^(34)^. Total amount of physical activity was expressed as Metabolic Equivalent Task (MET) minutes per week. MET-minutes were computed by multiplying MET by minutes of participation in moderate and vigorous intensity physical activity and in walking according to the IPAQ scoring protocol guidelines^(35)^.

### Statistical analyses

Descriptive statistics (e.g. means, standard deviations and percentages) were used to describe socio-demographic and socio-economic data. The measures of total physical activity were positively skewed, and due to the non-normal distribution of physical activity (MET-min), a logarithmic transformation (log PA) was performed to make the log physical activity data normal.

To assess the efficacy of the intervention, the outcome variables were examined using repeated measures ANCOVA (rANCOVA). Based on the available evidence, a set of explanatory variables were initially considered for adjustment before conducting rANCOVA, and the collinearity of the explanatory variables was examined^(36)^. Father’s education was significantly associated with mother’s education, and as such a decision was made to include mother’s education in the modelling as mothers’ healthy dietary behaviours may influence their children in promoting healthy eating practices. Finally, rANCOVA used to examine the group differences was adjusted for relevant covariates including age, sex, BMI *Z*-score, mother’s education level, physical activity, screen time and sleep duration. Bonferroni *post hoc* tests for adjustment for multiple comparisons were used to examine the pair-wise differences.

An rANCOVA was employed to examine the daily changes in fresh fruit, vegetable intake and carbonated soft drinks consumption. Interaction effects (group*wave) were examined to see whether the intervention group, compared to the control group, had differential effects on the changes in the outcome measures over time. When insignificant, the interaction term was dropped from the model to simplify the analysis. Outliers and other assumptions of the models were checked, and the model fit was assessed before finalising the models. All analyses were conducted using SPSS version 25. Significance levels were set at *P* ≤ 0·05.

## Results

Table 1 presents the baseline characteristics of the participants. A total of 320 students, 160 per group, (41·3 % girls) aged 13–17 years enrolled in this study. The level of mothers’ education was similar for both groups. Participants’ mean ages were 14·42 ± 1·15 and 14·18 ± 0·89 years in the intervention (34·4 % girls) and control schools (48·1 % girls), respectively, with a significant sex difference at the baseline. The majority of the participants also had a normal BMI range (intervention schools = 75 %; control schools = 72·5 %). Monthly family income was similar across the two groups. At baseline, the weekly physical activity was significantly lower in the control group compared to the intervention group, while students in the intervention group spent more screen time daily with a significant group difference (Table 1).

**Table 1:** Baseline characteristics of participants from the intervention and control schools in Dhaka City, Bangladesh

Characteristics	Intervention group (*n* 160)		Control group (*n* 160)		*P*-value
	Mean	sd	Mean	sd	
Age in years	14·42	1·15	14·18	0·89	0·040‡
	*n*	%	*n*	%	
Gender					
Boys	105	65·6	83	51·9	0·012§
Girls	55	34·4	77	48·1	
BMI					
Underweight	14	8·7	12	7·5	0·716§
Normal weight	120	75·0	116	72·5	
Overweight	20	12·5	27	16·9	
Obese	6	3·8	5	3·1	
BMI *Z*-scores	−0·37	1·34	−0·05	1·23	0·028‡
Mother education level					
Up to primary or equivalent	25	15·6	32	20·0	0·184§
Secondary (SSC) or equivalent	35	21·9	40	25·0	
Higher secondary (HSC) or equivalent	59	36·9	41	25·6	
Tertiary (graduation) or above	41	25·6	47	29·4	
	Median	IQR	Median	IQR	
Total physical activity MET-min/week†	999	570, 2193	522	247, 990	<0·001‖
Total screen time min/d*,†	308	228, 372	239	173, 332	<0·001‖
Sleep duration (h/d)	6·93	6·57, 8·00	7·29	6·57, 8·00	0·78‖

SSC, secondary school certificate; HSC , higher secondary certificate.

* A total screen-time measure was derived by taking the weighted average (i.e. (school-day screen time × 5) + (weekend-day screen time × 2)/7).

† Median (interquartile range (IQR)) was reported due to non-normal distribution of physical activity and screen time data.

‡ Based on *t* test.

§ Based on Chi-square test.

‖ Based on Mann–Whitney *U* test.

Table 2 displays the observed frequency of fresh fruit, vegetables and carbonated soft drink intake across different time points by the two groups.

**Table 2:** Observed mean values of fresh fruit, vegetables and carbonated soft drinks (times/d) intake across different time points by the two groups

Variables	Intervention		Control	
	Mean	sd	Mean	sd
Fruit				
Baseline	2·14	0·92	1·99	0·89
8 weeks	2·34	0·96	2·01	0·95
12 weeks	2·49	0·99	2·07	0·91
Changes (baseline to 12 weeks)*	0·35	1·36	0·08	1·09
Vegetables				
Baseline	2·37	1·41	1·71	1·00
8 weeks	3·05	1·33	1·87	1·23
12 weeks	3·24	1·30	1·77	1·16
Changes (baseline to 12 weeks)†	0·87	1·75	0·06	1·53
Carbonated soft drinks				
Baseline	3·11	1·14	2·85	1·06
8 weeks	2·56	1·28	3·01	1·11
12 weeks	2·48	1·41	2·88	1·20
Changes (baseline to 12 weeks)‡	−0·63	1·81	0·03	1·43

* *t* test, *P*-value: 0·052.

† *t* test, *P*-value: <0·001.

‡ *t* test, *P*-value: <0·001.

### Frequency of fresh fruit intake

**Fig. 2:**
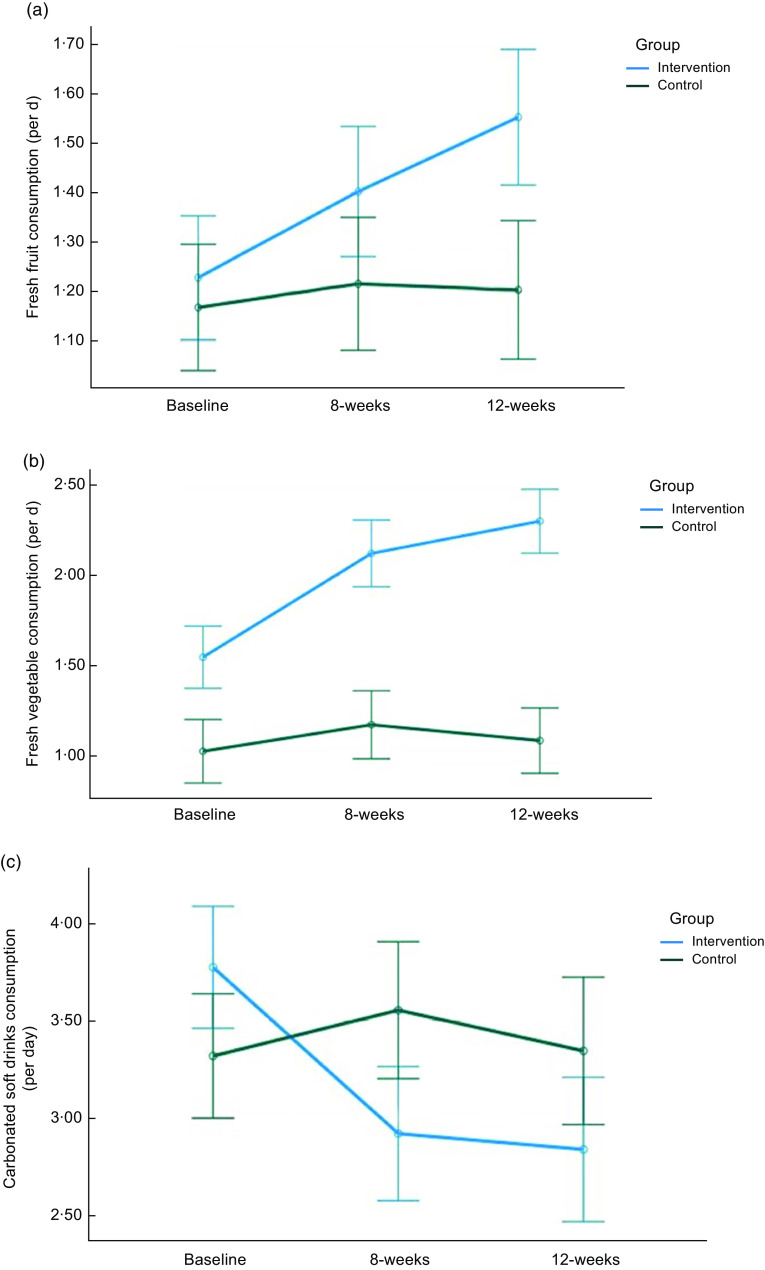
Average frequent intake of (a) fresh fruits, (b) fresh vegetables, and (c) carbonated soft drinks for intervention and control groups is displayed from baseline to the 12 weeks. The x-axis represents time points, ranging from baseline to the 12 weeks, while the y-axis indicates the daily mean consumption. This figure highlights changes in dietary habits over the course of the study and enables a comparison between the two groups

Both the intervention and control groups had significant improvements in the frequency of fruit intake from baseline to 12 weeks. We found a significant improvement in the daily frequency of fresh fruit intake between the intervention (26 %) and the control group (3 %) at 12 weeks [*F*(1,305) = 7·75, *P* = 0·006; partial *η* ^2^ = 0·025] (Table 3). The mean number of fruit intake per d was 1·7 in the intervention group at 12 weeks (Fig. 2(a)). Although not statistically significant, daily frequency of fresh fruit intake was higher in the intervention (14 %) than in the control groups (4 %) at 8 weeks compared to baseline. No significant interaction effects were observed for daily frequency of fresh fruit intake between groups over time (data not shown).

**Table 3:** Intervention effects on intake of fresh fruit, vegetables and carbonated soft drinks (times/d) of study participants across the two groups

Variables	*F*	df†	*P*-value	Partial *η* ^2^
Fruit				
Group	7·75	1, 305	0·006	0·025
Wave	0·08	1·95, 595	0·092	0·001
Vegetables				
Group	95·50	1, 305	<0·001	0·238
Wave	0·17	1·97, 602	0·84	0·001
Carbonated soft drinks				
Group	1·71	1, 305	0·193	0·006
Wave	0·33	1·92, 586	0·710	0·003
Group × Wave*	6·23	1·92, 586	0·002	0·047

† df, degrees of freedom.

* Group*wave interaction effect.

The ANCOVA model adjusted for sex, age, total physical activity, total screen time and BMI *Z*-score.

Frequency of fresh vegetable intake: changes in the frequency of fresh vegetable intake for adolescents are presented in Fig. 2(b). The intervention group reported a significantly higher proportion of fresh vegetable intake (37 % and 49 %) compared to the control group (14 % and 2 %) at 8 weeks and 12 weeks [*F*(1,305) = 95·50, *P* < 0·001; partial *η* ^2^ = 0·238] (Table 3). The mean number of vegetable intake per d was 2·2 and 2·4 in the intervention group at 8 weeks and 12 weeks, respectively (Fig. 2(b)). No significant interaction effects were found for the frequency of fresh vegetable intake between groups over time (data not shown).

Carbonated soft drink intake: a significant group by wave interaction was observed for the frequency of carbonated soft drink intake [*F*(1·92, 586) = 6·23, *P* = 0·002; partial *η* ^2^ = 0·047] (Table 3). Although there was no group difference, proportion of frequent carbonated soft drink intake was decreased in the intervention group (23 % and 25 %) at 8 weeks and 12 weeks (Fig. 2(c)), while it remained the same in the control group.

## Discussion

Using a cluster-randomised controlled trial, this study assessed the efficacy of an education intervention in increasing the frequent intake of fruit and vegetable and decreasing carbonated soft drink consumption among adolescents. Daily fresh vegetable and fruit intake was significantly higher in the intervention compared to the control group. In the intervention group, frequent intake of carbonated soft drinks had significantly decreased from baseline to 12 weeks.

Our intervention was effective in increasing the frequent intake of fruits from baseline after 12 weeks of intervention, which is consistent with the findings of a review that school-based interventions with 6 weeks to 5 months durations are effective in improvement of dietary behaviours (e.g. increasing fruit and/or vegetable intake) in the intervention group compared to the control group among adolescents^(37)^. The results are also in line with previous multi-component school-based interventions that were conducted in developing countries utilising a school-wide or health-promoting school approach with an intervention duration of 12 to 20 weeks, reported a significant increase in the frequent consumption of fruits among adolescents in the intervention group^(38,39)^. Studies in India were mainly focused on classroom activities with a length intervention period of 6 and 9 months to promote healthy eating behaviours (e.g. increasing frequency of fruit intake)^(17,40)^. In a study of Danish adolescents, Jørgensen *et al*.^(41)^ found that parental involvement significantly influences adolescents’ fruit and vegetable intake. Similarly, earlier interventions in developing countries have shown that parental involvement was an essential component to promote healthy eating practices at home using educational materials and parent–teacher association meetings^(17,38–40)^. Factors that possibly contributed to the present findings were that the intervention included not only the classroom approach but also involved outreach to the family via educational materials in promoting healthy eating practices for adolescents. This study also focused on interactive learning through group discussion with peers to enhance motivation for action and reinforce healthy eating behaviours, which might be a key component of the intervention package as found in studies from developed and developing countries^(22,23)^. On top of the classroom education lesson, take-home messages using easy-to-read educational materials might have also helped adolescents in the intervention group to recall and encourage them to eat more fruits.

In this study, a significant improvement was found in daily frequent intake of vegetables among adolescents after the completion of a 12-week classroom-based education intervention. Similarly, three multi-component school-based interventions in India reported a significant improvement in the intake of vegetables in the intervention group with a duration of 5 to 9 months^(23,38,40)^. Saraf *et al*.^(40)^ and Thakur *et al.* ^(38)^ both used a classroom-based education involving parents and educational materials to increase the frequent intake of vegetables and fruit among adolescents in the intervention group, as used in the present study. In a study from Italy, Egidio *et al.* identified that classroom education sessions improved children’s nutrition behaviour^(42)^. Additionally, the current study is consistent with systematic reviews which have proven to promote positive attitudes towards the healthy dietary intake of adolescents using the health-promoting framework or a whole school-wide approach^(21,43)^. A recent systematic review also concluded that classroom-based education intervention was beneficial in improving adolescents’ healthy dietary choices^(44)^. The majority of the school-based studies from this review had a randomised or cluster-randomised controlled trial including the intervention topics: food groups, food sources, food pyramid, balanced diet, fruits and vegetables, which were likely to be similar to our components. The result from this review found a strong parental influence on adolescents improving their knowledge, attitude and practice on vegetable intake. It was notable that adolescents in the intervention group of our study increased the frequent intake of fresh vegetables over time, and this may be due to the parental healthy eating practices at home that are more likely to foster healthy eating patterns in their child.

After the intervention, significant interaction effects were found, and the daily frequent intake of carbonated soft drinks decreased from baseline in the intervention group. Singh *et al*. implemented a school-based intervention with Dutch adolescents which offered education sessions as a nutrition improvement tool and reduced the frequent intake of sugary soft drinks in the intervention to the control group after 8- and 12-month follow-up^(45)^. On the contrary, Haerens *et al.* ^(46)^ found no intervention effects on carbonated soft drink consumption among adolescents after a 12-month intervention. It was notable that both earlier studies delivered class-based education sessions, which is similar to our intervention component to promote healthy eating practices among adolescents. The adolescents in our intervention group reported a lower frequent intake of carbonated soft drinks over time. One possible explanation for this reduction might be the use of educational materials and interactive classroom activities that motivate and reinforce them to reduce the frequent intake of carbonated soft drink consumption as reported elsewhere^(22,23)^.

The study has several strengths, including the use of a cluster-randomised controlled trial design and the inclusion of classroom-based education with interactive intervention components. This intervention used the WHO’s Health Promoting Schools framework or a whole school approach, which was implemented within the school curriculum of Bangladesh. Some limitations of the present study include assessment of outcomes using unvalidated instruments and self-report of the outcomes. Self-report may lead to overreporting of healthy behaviours (e.g. intake of fruits and vegetables) and an underreporting of unhealthy behaviours (e.g. soft drinks consumption). Our dietary measures only provide the frequency of daily intake of fruit and vegetables with no reference to the amount of consumption, which is one of the main limitations of this study.

However, a recent meta-analysis reported that the GSHS are nationally representative self-administered questionnaires that provide valuable insight into the frequency of fruit, vegetable, carbonated soft drink and fast-food consumption among school-going adolescents (aged 12–17 years)^(14)^. Physical activity was assessed using IPAQ-A, which was not validated in Bangladeshi adolescents. In the present study, the modelling did not take into account the clustering of students within schools as data consisting of fewer than ten clusters could potentially bias the results^(47)^. Another limitation is that the intervention was interrupted by 1 month at 8 weeks due to the Ramadan break (Ramadan is a warm, community celebration for practising Muslims that involves a month of fasting, worship and feasting). Additionally, our intervention of 12-week duration might have been early to evaluate the changes in dietary behaviours. A recent systematic review^(48)^ reported that a short-term (e.g. up to 6 months) intervention could be successful in promoting physical activity behaviours among Asian children and adolescents. However, we were unable to design the trial beyond 12 weeks due to logistic constraints. Moreover, all participants were from public and semi-public schools in urban settings, which may not represent other schools that might have different cultures, socio-economic status and school policies in promoting healthy eating behaviours.

### Conclusion

Our classroom-based education intervention in a school setting may provide a practical and effective approach that has the potential to promote healthy eating behaviours of Bangladeshi adolescents. This study demonstrated better improvements in the frequent intake of fresh fruit and vegetables among adolescents in the intervention group. Frequent intake of carbonated soft drinks is also decreased among the intervention group over time. The findings of our study can inform designing of experimental studies, which can be implemented in different educational settings across rural and urban areas to promote healthy eating behaviours of school children in Bangladesh. In addition, our intervention components could be embedded into the Bangladesh national curriculum through interactive activities and have the potential for greater adaptation across the country.

## Supplementary material

For supplementary material accompanying this paper visit https://doi.org/10.1017/S1368980023002094
